# The Pancreas Is Altered by *In Utero* Androgen Exposure: Implications for Clinical Conditions Such as Polycystic Ovary Syndrome (PCOS)

**DOI:** 10.1371/journal.pone.0056263

**Published:** 2013-02-15

**Authors:** Mick Rae, Cathal Grace, Kirsten Hogg, Lisa Marie Wilson, Sophie L. McHaffie, Seshadri Ramaswamy, Janis MacCallum, Fiona Connolly, Alan S. McNeilly, Colin Duncan

**Affiliations:** 1 School of Life, Sport and Social Sciences, Edinburgh Napier University, Edinburgh, United Kingdom; 2 MRC Centre for Reproductive Health, The Queens Medical Research Institute, The University of Edinburgh, Edinburgh, United Kingdom; Imperial College London, United Kingdom

## Abstract

Using an ovine model of polycystic ovary syndrome (PCOS), (pregnant ewes injected with testosterone propionate (TP) (100 mg twice weekly) from day (d)62 to d102 of d147 gestation (maternal injection – MI-TP)), we previously reported female offspring with normal glucose tolerance but hyperinsulinemia. We therefore examined insulin signalling and pancreatic morphology in these offspring using quantitative (Q) RT-PCR and western blotting. In addition the fetal pancreatic responses to MI-TP, and androgenic and estrogenic contributions to such responses (direct fetal injection (FI) of TP (20 mg) or diethylstilbestrol (DES) (20 mg) at d62 and d82 gestation) were assessed at d90 gestation. Fetal plasma was assayed for insulin, testosterone and estradiol, pancreatic tissue was cultured, and expression of key β-cell developmental genes was assessed by QRT-PCR. In female d62MI-TP offspring insulin signalling was unaltered but there was a pancreatic phenotype with increased numbers of β-cells (*P*<0.05). The fetal pancreas expressed androgen receptors in islets and genes involved in β-cell development and function (*PDX1, IGF1R, INSR* and *INS*) were up-regulated in female fetuses after d62MI-TP treatment (*P*<0.05–0.01). In addition the d62MI-TP pancreas showed increased insulin secretion under euglycaemic conditions (*P*<0.05) *in vitro*. The same effects were not seen in the male fetal pancreas or when MI-TP was started at d30, before the male programming window. As d62MI-TP increased both fetal plasma testosterone (*P*<0.05) and estradiol concentrations (P<0.05) we assessed the relative contribution of androgens and estrogens. FI-TP (commencing d62) (not FI-DES treatment) caused elevated basal insulin secretion *in vitro* and the genes altered by d62MI-TP treatment were similarly altered by FI-TP but not FI-DES. In conclusion, androgen over-exposure alters fetal pancreatic development and β-cell numbers in offspring. These data suggest that that there may be a primary pancreatic phenotype in models of PCOS, and that there may be a distinct male and female pancreas.

## Introduction

The steroidal milieu of pregnancy may have diverse and profound effects upon the physiology of the developing fetus, some of which may persist into adulthood, conferring lifelong health consequences. Animal models developed to investigate such legacies of the fetal environment that have clinical implications have recently focussed upon polycystic ovary syndrome (PCOS).

PCOS is common; some twenty percent of women have polycystic ovaries, and approximately one third of these have additional clinical and biochemical features of PCOS [1]. In addition to ovarian and reproductive hormonal phenotypes, women with PCOS are at increased risk of altered metabolic parameters including hyperinsulinemia and the development of insulin resistance (IR) [2], [3].

Despite intense study, there remains a lack of clear identification of origins and developmental pathways of this syndrome. Nonetheless, numerous studies indicate that the prenatal environment may play a fundamental role in the development of PCOS, subsequently realised in adult life. Animal models designed to study potential origins of PCOS have commonly utilized the single intervention of androgen overexposure during *in utero* life [4], [5] to recreate many of the symptoms of PCOS in offspring, notably in rodents [6], monkeys [7] and sheep [8], [9], [10]. The legacy effects of increased androgens on female fetuses seen in those models seem to be recapitulated by experiments of nature in women [11].

As IR is associated with compensatory increased insulin secretion [3], hyperinsulinemia associated with PCOS is usually considered a consequence of IR. However, rodent studies provide evidence that prenatal androgen exposure causes alterations in pancreatic β-cell glucose sensitivity [6] and our previous work demonstrated altered adult insulin secretion in animals treated prenatally with androgens, in the absence of glucose intolerance [9]. We hypothesized that there may be an underlying primary pancreatic alteration that precedes functional pancreatic changes in response to IR.

Hence in this study we have further examined insulin signalling at a molecular level in these animals, examined pancreatic α and β-cell content, and assessed the potential for androgenic-programming of fetal β-cell function and pancreatic development. We include male fetal data sets where pertinent to address the possibility that such steroidal manipulations create a phenotypically male pancreas in females. Finally, due to potential placental/maternal metabolism of testosterone, there remains the issue as to whether or not endpoints measured result from androgenic or estrogenic stimuli, and to resolve this we have utilised a direct fetal-treatment model where we directly administered testosterone propionate (TP) to the fetus and, in terms of key gene expression changes and *in vitro* β-cell function, compared effects of this route of administration with both maternal injection (MI) of TP, and direct fetal injection (FI) of the potent estradiol agonist diethylstilbesterol (DES).

We hypothesise that prenatal exposure to excess androgens results in altered female pancreatic β-cell function in fetal life, and pancreatic function in adult female offspring, with the implication that pancreatic β-cell dysfunction may be a primary effect of prenatal androgen overexposure.

## Materials and Methods

### Ethics Statement

All studies were approved by the UK Home Office (conducted under approved Project Licence (PIL 60/3744) after review by the University of Edinburgh Animal Research Ethics Committee.

### Animals

Scottish Greyface ewes were fed to achieve comparable body condition prior to estrous cycle synchronization. After synchronized mating, animals were allocated randomly to two experimental groups. The experimental groups were given twice weekly injections of TP (100 mg dissolved in vegetable oil) or vehicle (vegetable oil) from day (d)30 or d62 gestation until d102 gestation or sacrifice (term is 147 days). Animals carrying to term were conventionally managed after this treatment period. In all cases euthanasia was achieved by barbiturate overdose and numbers are indicated in the figure legends.

Fetal tissue and plasma was collected at d90 gestation. Actual numbers of fetuses used in each analysis are specified in figure legends; differences in numbers of fetuses used are attributable to constraints of tissue available for each analysis. Furthermore, due to differences in glucose handling between singleton and twin fetuses during gestation [12], in all analyses only fetuses from twin pregnancies were analyzed. In the case of animals that carried to term, no differences in birth weight associated with any treatment regimen were evident, and offspring were reared and managed conventionally (weaned at 3 months) until sacrifice (barbiturate overdose) at 11 months postnatal age, when tissues were recovered 15 min after intravenous glucose (500 mg/ml in 20 ml) bolus administration.

Due to practical and economic constraints only female offspring were reared through to adulthood. In the case of direct FI studies of steroids, anaesthesia was induced by initial sedation (10 mg Xylazine; i.m., ‘Rompun’, Bayor plc Animal Health Division, UK) then administration of 2 mg/kg ketamine (i.v., Keteset, Fort Dodge Animal Health, UK). Under aseptic surgical conditions fetuses were injected with 200 µl vehicle alone, 20 mg TP, or 20 mg DES dissolved in vehicle oil via. a 20G Quinke spinal needle (BD Biosciences, UK), via ultrasound guidance at day 62 and 82 of gestation to coincide and replicate via depot injection the exposure time frame of the d62 MI-TP cohort. Immediately after completion of this surgical procedure all ewes were administered prophylactic antibiotics (1 ml/25 kg; Streptacare, Animalcare Ltd, UK) and the animals were sacrificed on gestational d90.

### Tissue Collection

Two weeks prior to sacrifice the female MI-TP offspring had a thigh muscle biopsy taken under local anaesthesia (using 2% Lidocaine) before and 15 min after i.v. glucose administration (500 mg/ml in 20 ml). This was snap frozen and stored at −80 C for subsequent protein extraction and western blotting. At sacrifice pancreatic tissue was collected and fixed in Bouins solution for 24 h, then rinsed, transferred to 70% ethanol and embedded in paraffin wax for histological analyses. In addition skeletal muscle, visceral fat from the omentum, and liver biopsies were collected, snap frozen and stored at −80 C for subsequent molecular analysis. In the case of pregnant ewes, fetal blood was collected and fetuses dissected immediately post-sacrifice. The pancreas of each fetus was removed, and divided into two/three approximately equal portions. One piece was fixed and processed for histological analysis as described above, the second piece was immediately snap frozen and stored at −80 C for subsequent mRNA extraction, and in a subset of animals the third piece immediately processed for tissue culture as described below.

### Examination of Gene Expression by Quantitative (Q) RT-PCR

For RNA extraction, frozen adult tissue samples were homogenized with 1 ml TRI-reagent (Sigma Aldrich, UK) following the manufacturer’s instructions and the RNA was dissolved in 20 µl RNase-free water. Frozen fetal pancreas was homogenised in RLT buffer (including 2-mercaptoethanol, 1% v/v) (Qiagen, Crawley, UK) and RNA was extracted using RNeasy minispin columns following the manufacturer’s protocols, including on-column DNaseI digestion (Qiagen, Crawley, UK). RNA concentrations were determined using a NanoDrop 1000 spectrophotometer (Thermo Fisher Scientific, Loughborough, UK), with gel electrophoresis used to monitor quality. Complimentary DNA (cDNA) was synthesized according to kit manufacturer protocols (Applied Biosystems, California, USA).

Previously published and validated primer pairs were used where possible. This included *GAPDH* and *AR* [9] and *PDX1, IGF1, IGF2, IGFR1, IGFR2*, *SLC2A2* (*GLUT2*) and *INSR* [13]. Ovine *INS* primers were designed and validated by Primer design Ltd (fwd: CCAGCGGGAAATCAAGAGAGA, rev: CCTAGGGAGCTGGTCACTT). Otherwise primers were designed (Primer3 version 0.4) and checked for specificity using Basic Local Alignment Search Tool (BLAST). Primers were synthesized (Eurofins MWG Operon, Ebersberg, Germany) and validated as previously described [9], [10]. These primer pairs included: *IRS1* (fwd: ATCATCAACCCCATCAGACG, rev: GAGTTTGCCACTACCGCTCT), *SLC2A1* (*GLUT1)* (fwd: TGCTGAGCGTCATCTTCATC, rev: GGCTCTCCTCCTTCATCTCC), *SLC2A4* (*GLUT4)* (fwd: ACCTTATGGCCACTCCTCCT, rev: CTCAGCCAACACCTCAGACA) and *LEP* (fwd: ATC TCA CAC ACG CAG TCC GT, rev CCA GCA GGT GGA GAA GGT C). Gene expression in adult tissues was investigated using a previously described protocol [14]. Data was normalized and derived as previously described using *GAPDH* as a reference gene [9], [10].

For fetal pancreas samples SYBR Green QRT-PCR was prepared with Sensimix (Bioline UK), primer pairs (0.5 µM), cDNA (1 µl) and nuclease free water. Reactions were carried out in triplicate using a StepOne Plus platform (Applied Biosystems, UK). Melting curve analysis revealed a single amplicon in all cases. Gene expression relative to *GAPDH* was quantified using the 2-ΔΔCt method in the case of MI experimental samples after identification of *GAPDH* as a stable housekeeping gene (Genorm analysis (Primer Design Ltd) of 12 ovine reference genes). However, in the case of FI samples, which encompassed FI-vehicle, FI-TP and FI-DES treatments, Genorm analysis (PrimerDesign Ltd, UK) revealed that an additional two housekeeping genes were required to give robust housekeeping stability, hence the geometric mean of *GAPDH*, *ATP5B* and *ACTB* was used as the normalization reference. Ovine placenta was used as a positive control, and negative controls consisted of an RT-ve and a template negative reaction.

### Western Blotting

In the case of western blotting studies, five samples were selected randomly from each group of 11-month old females (MI-TP exposed). Protein extraction and pre-clearing was based upon previously published protocols [15]. Protein content was assayed (Bio-Rad, Hemel Hempstead, UK) and, after heat denaturation, samples (10 µg total protein) underwent electrophoresis (5% stack/12% polyacrylamide resolving) and blotting onto Hybond–P membranes (GE Healthcare, UK) following the manufacturer’s protocols. Membranes were blocked in 5% defatted milk solution in Tris Buffered Saline (TBS)/0.1% Tween (TBST) for 1 h at room temperature, then probed with primary antibodies raised against total ERK1/2, phospho-ERK1/2 (Thr^202^/Tyr^204^), total AKT, phospho-AKT (Ser^473^) (Cell Signalling Technologies Inc. USA) and β-actin (Sigma Aldrich, UK).

After washing the secondary antibody (chicken anti-rabbit peroxidise; Santa Cruz Biotechnology Inc. California USA), diluted 1∶2000 in 1% milk/TBST was applied for 1 h before washing, application of ECL Plus Western Blot Detection System (GE Healthcare, UK) and exposure to X-ray Film (Super RX Film, FujiFilm Medical Systems UK). Images were then quantified by densitometry (GS-700, Bio-Rad Laboratories Ltd, Hertfordshire, UK) and normalized optical density measured using α-tubulin as the loading control (Image J Image Processing and Analysis in Java software (http://rsbweb.nih.gov/ij/)). The ratio of phosphorylated protein to total protein was then calculated.

### Fetal Pancreatic Tissue Culture

Pancreatic tissue was dissected under aseptic conditions and resuspended in HEPES buffered saline solution (HBSS) containing 3 mg/ml collagenase (Sigma Aldrich, Poole, UK), and incubated for 20 min at 37C with gentle agitation. After passing through a 19G needle 3 times, cell suspensions were centrifuged (600 g, 10 min, room temperature) and the collagenase solution replaced with 2 ml HBSS, and the centrifugation step repeated, prior to resuspension in 1 ml culture medium (Dulbecco’s Modified Eagle Medium (DMEM), 2 mM *L*-glutamine, 100 U/ml penicillin, 0.1 mg/ml streptomycin, 0.25% bovine serum albumin (BSA), pH 7.4 (final glucose concentration 5.5 mM) (Sigma-Aldrich).

Aliquots (100 µl) of cell suspension were added to 300 µl of culture medium plus treatments (basal glucose/euglycaemic (5.5 mM), high glucose/hyperglycaemic (20 mM) and incubated for 3 h, with gentle agitation, at 37C, 5% CO_2_, in sterile tubes with vented lids. At the end of the incubation period tubes were centrifuged (600 g, 15 min), and supernatants removed and frozen at −20C until insulin measurement. Cell pellets were resuspended and washed twice in 1 ml PBS, pH 7.4 then re-pelleted by centrifugation and stored at −20C until protein determination.

Pancreatic cell pellets were resuspended in lysis solution (50 mM Tris-HCl, pH 8.0, 150 mM NaCl, 0.1% Triton-X-100) and sonicated for 2 min, followed by one minute incubation on ice, then a further 2 min sonication on ice. Protein was measured as described above. In pilot experiments glucose treatments were replicated using mannitol as a glucose substitute to ensure effects of glucose were specific and not due to osmotic change. Mannitol (20 mM) did not alter insulin secretion. In the case of FI tissue only basal (5.5 mM) glucose was examined.

### Hormone Determinations

Insulin in tissue culture supernatants and plasma samples was measured using ELISA kits for ovine insulin (ALPCO, Salem, NH). Data were collected using a Dynex Technologies Revelation 4.25 plate reader (dual wavelength detection) utilizing cubic spline curve fit and detection wavelength of 450 nm and a reference wavelength of 630 nm as per manufacturer’s instructions. All samples were assayed in duplicate, and on a single plate per experiment, with an intra-assay coefficient of variation of 4.5% and sensitivity of 0.14 ng/ml.

Steroid assays were performed on solvent–extracted plasma samples. Briefly, 100 µl serum for testosterone, and 200 µl serum in the case of estradiol assays was mixed with 2 ml diethyl ether (VWR International) and vortexed for 3 min. Solvent and aqueous phases were separated via freezing, and the solvent phase evaporated. Tubes containing the dried extract were re-constituted in phosphate gelatine buffer saline (PGBS) assay buffer. Testosterone measurement was performed in duplicate using a standard in-house radioimmunoassay [9], [16], where the intra-assay coefficient of variation was <10%, and assay sensitivity 12 pg/ml. Estradiol concentrations were measured using the MAIA Oestradiol ELISA Kit (37001, Inverness Medical UK, Cheshire, UK) as per the manufacturer’s instructions as described previously [9], where the intra and inter-CVs were 6% and 9%, respectively and assay sensitivity was 0.3 pg/ml.

### Immunohistochemistry

Tissue sections (5 µm) were mounted onto positively charged slides (Superfrost Plus Gold, ThermoScientific, Epsom, UK) and dried overnight at 40C. After clearing in xylene and rehydration through graded alcohols, antigen retrieval was performed by microwaving (750W) for 5 min three times in 0.1 M sodium citrate buffer, pH 6.0, with 1 min between each heating step and 20 min rest period at the end of this process. Slides were then incubated in 3% H_2_O_2_ followed by two further 5 min washes in PBS. After blocking with 2.5% horse serum (Vector laboratories, Peterborough, UK), the sections were incubated with primary mouse anti-insulin monoclonal antibody (Abcam, Cambridge, UK) at a dilution of 1 in 500 (10 µg/ml), primary rabbit anti-glucagon antiserum (Millipore UK) (1 in 500 dilution) or rabbit anti-AR antiserum (1 in 50 dilution) (Santa Cruz, Santa Cruz, CA), overnight at 4C. Negative controls consisted of mouse IgG or non-immune rabbit serum at an identical concentration to that of the primary antiserum.

Slides were then washed twice for 5 min in PBS containing 0.1% Tween-20 (PBST) and treated with biotin-conjugated secondary antibody (pan-specific anti-mouse and rabbit, Vector laboratories) for 30 min. After two further washes in PBST, ABC complex (Vector laboratories) was applied to sections for 30 min. After a further two washes in PBST 3,3-diaminobenzidine (Vector laboratories) was used to visualize bound antibody. The slides were then washed, counterstained in haematoxylin (Sigma-Aldrich), dehydrated, cleared in xylene and mounted in Pertex (Vector laboratories). Initial pilot studies were conducted to determine numbers of sections and fields of observation to give accurate, representative mean counts per sample. As a result of this pilot analysis, three sections of each fetal tissue, and five sections per adult tissue, in both cases cut 100 µm apart, were quantified.

Slides were digitally captured using Spot32 (Diagnostic Instruments Inc., Sterling Heights, MI, USA). To determine β-cell number, five random fields were examined in each section using an eyepiece grid, all positively stained cells counted and results were expressed as numbers of β-cells per mm^2^. In adult tissue islet size was determined via a calibrated eye-piece grid, measuring the area of 10 islets per section, in 5 sections per animal.

In studies of co-localization of AR and insulin, sections were processed as described above prior to being incubated with AR-specific antibody (Santa Cruz, Santa Cruz, CA) at a concentration of 1 in 50 in 2.5% goat serum overnight at 4C. After washing, the sections were incubated with fluorescently labelled secondary antibody (Alexafluor 633, goat-anti-rabbit) at a concentration of 1 in 200 in PBS, pH 7.4, for 30 min and washed twice in PBST and once in PBS. Sections were then re-blocked in 2.5% goat serum and incubated with insulin antiserum at a concentration of 1 in 500 overnight at 4C. After washing, the sections were incubated with labelled secondary antibody (Alexafluor 488, goat-anti-mouse) at a concentration of 1 in 200, for 30 min. After washing, the sections were mounted in Mowoil (Calbiochem, Nottingham, UK), stored overnight at 4C and visualized using a Zeiss LSM 500 Meta system confocal microscope. Negative controls were as described for conventional immunohistochemistry.

### Statistical Analysis

Statistical analyses were performed with Graph Pad Prism v4.0 (GraphPad Software Inc., San Diego, CA) and values of *P*<0.05 regarded as significant. Student’s t-test (where applicable) and one-way ANOVA with Tukey’s post hoc testing was applied. Where data showed a non-normal distribution log (base10) transformation was applied prior to analysis.

## Results

### Peripheral Insulin Signalling in 11 Month Old Female Offspring from d62MI-TP Experimental Pregnancies

**Figure 1:**
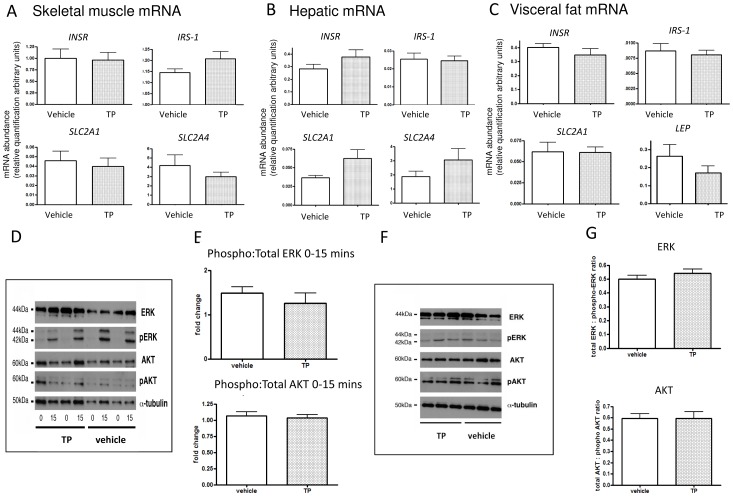
Molecular determination of skeletal muscle and hepatic insulin signalling in d62MI-TP 11 month old female offspring. Gene expression (Panels A–C, vehicle treated n = 5; TP treated n = 9) and protein phosphorylation (Panels D and F vehicle treated n = 5; TP treated n = 5) and its quantification (Panels E and G) relevant to insulin signalling in muscle, liver and visceral fat was analyzed in early adult female offspring from MI-TP pregnancies. No significant alterations in expression of key insulin signalling and reception genes were evident in either tissue associated with prenatal treatment. Panel D shows representative western blots of insulin signalling proteins (MAPK and AKT pathways) in muscle biopsies before and after glucose bolus and Panel F shows these pathways in muscle at sacrifice two weeks later after glucose bolus from the aforementioned pregnancies. Quantified data from these western blots in shown in Panels E and G – there were no differences in ratios of total : phosphorylated ERK or AKT associated with prenatal treatment.

As regards mRNA expression of key genes involved in insulin signalling, *in utero* d62MI-TP exposure had no significant effects in either muscle (Fig. 1A), liver (Fig. 1B) or visceral fat (Fig. 1C). Analyses of downstream insulin receptor signalling in skeletal muscle biopsies before and after glucose administration (Fig. 1D, representative blot)), or at sacrifice after a glucose bolus (Fig. 1F, representative blot), showed no alterations between *in utero* vehicle treated and d62MI-TP exposed animals in terms of total : phosphorylated ERK or AKT ratios (Fig. 1E, G quantified blots).

### Pancreatic Morphology of 11 Month Old Adult Offspring from MI-TP Experimental Pregnancies

**Figure 2:**
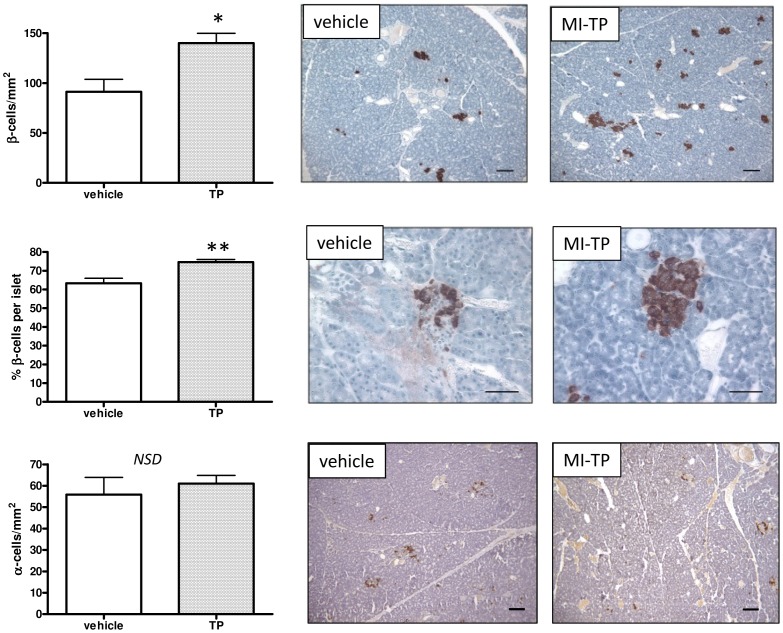
The effect of TP administration on adult endocrine pancreas. Female adult α- and β-cell content from vehicle and MI-TP treated pregnancies was assessed by immunohistochemistry. Prenatal TP treatment was associated with significantly increased total β-cell numbers at 11 months postnatal age in MI-TP offspring (n = 8) (*P*<0.05), which in turn was associated with increased numbers of β-cells per islet (*P*<0.01), as compared to vehicle treated offspring (n = 5). There were no effects of prenatal treatment as regards α-cell content. Photomicrographs are representative sections of adult pancreas from vehicle and MI-TP treated pregnancies (scale bars represent 50 µm).

We therefore assessed the effect of MI-TP treatment from d62 gestation on the morphology of the endocrine pancreas in terms of the numbers of α and β-cells. There was a significant increase in the total number of β-cells in prenatally d62 MI-TP-treated animals (*P*<0.05) (Fig. 2). Whilst there was no significant difference in islet size, there was a trend towards increased islet size (*P* = 0.07) (not shown), and a significant increase in the numbers of β-cells per islet (*P*<0.01) (Fig. 2). This was not associated with any change in the numbers of α-cells in the pancreatic islets (Fig. 2).

### Effects of d62 MI-TP Treatment on Fetal Pancreatic Morphology

**Figure 3:**
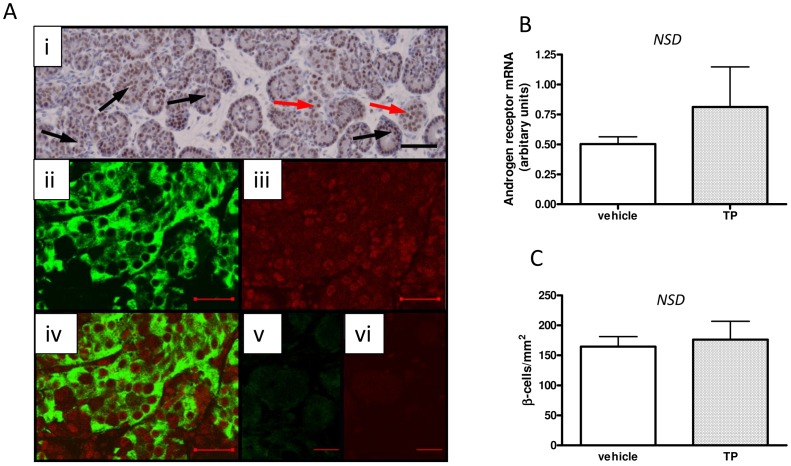
Ovine fetal pancreatic androgen receptor localization, *AR* expression and β-cell content. Panel A: (i) Fetal pancreas showing nuclear AR protein (brown) in multiple cell types including glandular tissue (black arrows) and presumed islets (red arrows). (**ii**) Fluorescent immunohistochemical staining for insulin (green) in a (vehicle) d90 fetal pancreas. (**iii**) Fluorescent immunohistochemical staining for AR (red) in a (vehicle) d90 fetal pancreas and (**iv**) represents a dual merge of insulin (green) and AR (red) confirming that β-cells express AR. (**v**) and (**vi**) show negative controls for insulin and androgen receptor respectively. Scale bar is 100 µm (**i**) and 25 µm (**ii–vi**). MI-TP had no effect on either *AR* abundance (n = 5, n = 8, vehicle and TP fetuses respectively) (Panel B) or fetal β-cell numbers (Panel C).

As these data suggested that maternal TP exposure in pregnancy results in an early pancreatic phenotype we assessed the fetal pancreas at the time of maternal androgenization. However, as the endocrine pancreas represents only a small percentage of total pancreatic tissue, and due to the difficulties associated with dissection of fetal pancreatic tissue, pancreatic weight is not a reliable measure [17] and as such we initially focused on immunohistochemical assessment of the developing fetal pancreas at d90 gestation. Androgen receptors were immunolocalized to multiple cell types in the fetal pancreas before d90 gestation (Fig. 3Ai). Dual-fluorescent immunohistochemistry confirmed that β-cells express AR by co-localizing AR and insulin in the same tissue sections (Fig. 3Aii–vi). This highlights that the fetal pancreatic cells with the potential to directly respond to androgens included the insulin secreting islet β-cells. However, there was no significant difference in the expression of AR (Fig. 3B) or the number of insulin-positive β-cells at d90 (Figure 3C) of gestation between tissues from female fetuses after maternal exposure to TP when compared to vehicle controls.

### Effects of Androgens on the Expression of Key Fetal Pancreatic Genes *in vivo*

**Figure 4:**
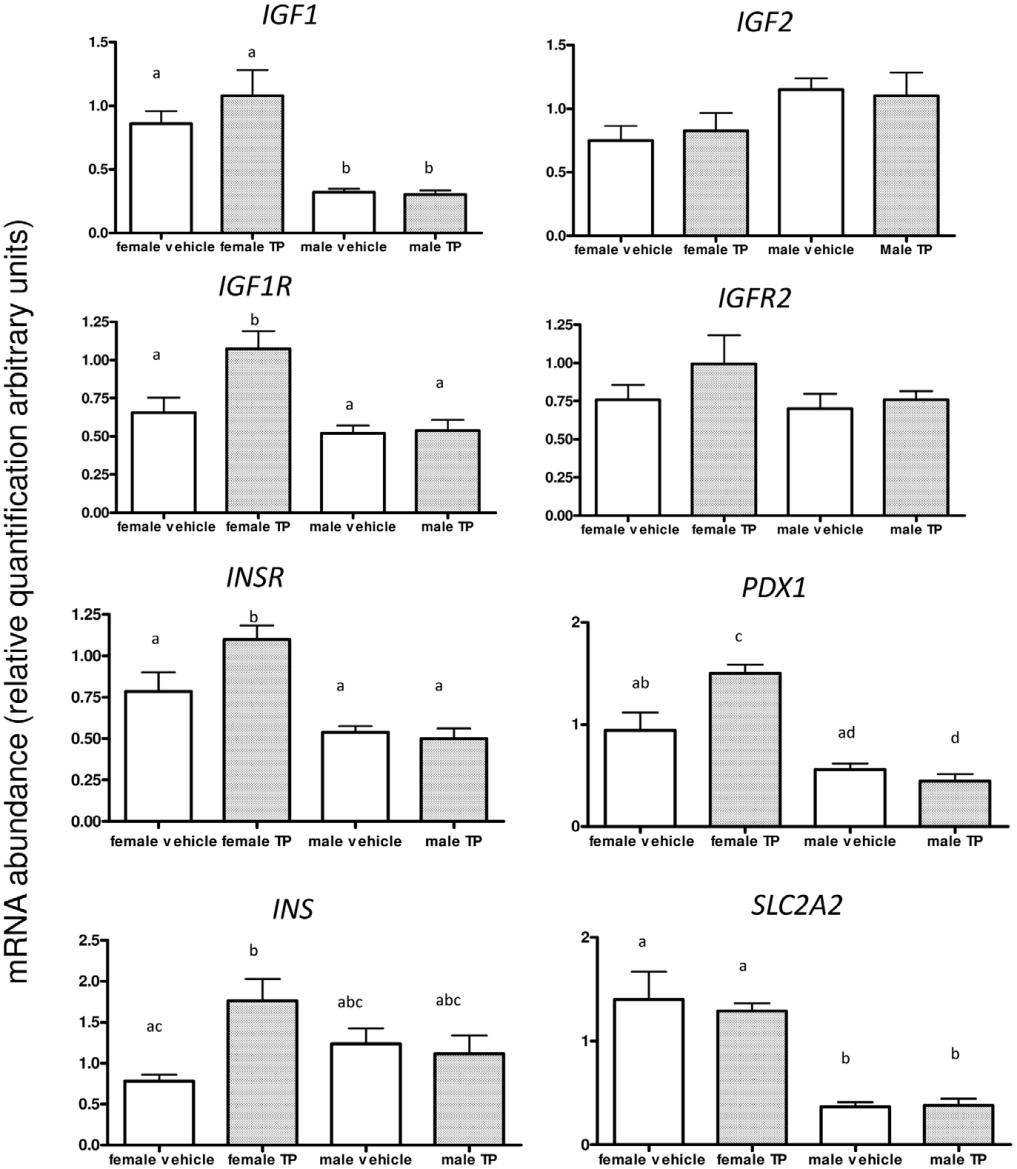
Fetal pancreatic expression of key developmental genes and *in vitro* insulin secretion is dependent upon fetal sex and timing of exposure to excess androgens. **A:** ***Female fetuses***: At d90, (vehicle n = 5, MI-TP n = 8), d62 MI-TP significantly increased expression of *IGF1R* (*P*<0.05), *PDX1* (*P*<0.01), *INSR* (*P*<0.05) and *INS* (*P*<0.05) mRNA. ***Male fetuses***: Whilst there were differences in expression attributable to the sex of the fetuses (see results section for full description), there were no differences in expression of any genes studied attributable to *in utero* treatment in male fetuses studied at d90 (vehicle n = 6, MI-TP n = 6) of gestation. Different superscripts denote significant differences (P<0.01–0.05, see results sections for full details).

We next investigated the expression of pancreatic genes pertinent to β-cell replication and function (Fig. 4). In female fetuses at d90 of gestation, d62 MI-TP administration did not significantly alter expression of *IGF1*, *IGF2*, *IGF2R* or *SLC2A2* (Fig. 4). However in female fetuses pancreatic expression of *IGF1R* (*P*<0.05), *INSR* (*P*<0.05), *PDX1* (*P*<0.01) and *INS* (*P*<0.05) were up-regulated by maternal TP administration from d62 (Fig. 4). Of particular interest was that maternal androgen treatment had no effect on the expression of these candidate genes in male fetuses. Furthermore there were differences in pancreatic gene expression between the control female and control male tissues (Fig. 4). Both pancreatic *IGF1* (*P*<0.01) and *SLC2A2* (*P*<0.001) had significantly higher levels of expression in female fetuses than that observed in male fetuses (Fig. 4).

### Effects of Maternal Androgenization from d30 on Fetal Pancreatic Gene Expression

We then studied the effect of earlier androgenization on the expression of key developmental genes in the fetal pancreas. The effects of maternal androgens administered from d30 gestation (d30MI-TP), which is before the sex determination window and results in masculinization of the external genitalia, are shown in Table 1. D30 MI-TP administration had no apparent effects on any gene measured in female fetuses apart from *IGF1R* expression that was elevated in a similar fashion to that observed in the d62 treated cohort (Table 1).

**Table 1:** Summary comparison of mRNA responses in female fetuses exposed to TP from d60 compared to female fetuses exposed to TP from d30 of gestation.

	Female	Female
	D62–90	D30–90
Gene	Vehicle *vs* TP	Vehicle *vs* TP
*INSR*	0.78±0.11 *vs* 1.09±0.08*	0.58±0.08 *vs* 0.72±0.09
*INS*	1.13±0.35 *vs* 1.76±0.26*	1.36±0.21 *vs* 1.49±0.10
*PDX1*	0.94±0.17 *vs* 1.50±0.08**	1.01±0.13 *vs* 1.29±0.05
*IGF1R*	0.65±0.17 *vs* 1.08±0.11*	0.46±0.03 *vs* 0.81±0.14*

D30 MI-TP (n = 6 vehicle, n = 6 TP) caused increased IGF1R expression in a similar fashion to that observed in female fetuses exposed to MI-TP from d62, but in the case of the other genes measured, there was no response to treatment from d30 in female fetuses, similar to the lack of response observed in d62 MI-TP male fetuses (see Figure 4). Asterisks denote significant difference between vehicle and TP treated *P<0.05;

** P<0.01.

### Effects on Androgen on Fetal Pancreatic Endocrine Function

Unfortunately we did not measure fetal glucose concentrations to allow full assessment of pancreatic function *in vivo*. There were no significant differences in fetal plasma insulin concentrations after maternal androgenisation (Table 2). However we were able to assess the functional consequences of maternal androgen administration on the endocrine function of the female fetal pancreas *in vitro*. Pancreatic tissue from vehicle and d62MI-TP groups was enzymatically digested and cultured in the presence of 5.5 mM and 20 mM glucose (Table 2). Although pancreatic tissue from both experimental groups secreted insulin only tissue from control fetuses was capable of mounting a significant increase in insulin secretion in response to 20 mM glucose (*P*<0.05 compared to 5.5 mM glucose). When exposed to 5.5 mM glucose, pancreatic tissue from MI-TP female fetuses had a comparable insulin response to that of vehicle treated tissue cultured in the presence of 20 mM glucose, thus giving rise to a significantly reduced glucose stimulation index (SI) [18] when compared to control tissue.

**Table 2:** *In vitro* glucose stimulated insulin secretion and plasma insulin from vehicle and d62 MI-TP fetuses at d90 gestation.

Female fetuses	Vehicle (*in vivo*)	D62–D90 TP *(in vivo*)	
*In vitro*	Insulin secreted	Insulin secreted	
	ng/mg tissue (SI)	ng/mg tissue (SI)	Significance
**Basal (5.5 mM glucose)**	0.4±0.03 (1.75±0.16)	1.30±0.32 (0.89±0.19#)	P<0.05
**Maximal (20 mM glucose)**	0.71±0.1*	1.22±0.42	P<0.05
***In vivo***	**Plasma Insulin ng/ml**	**Plasma insulin ng/ml**	
	0.83±0.19	0.48±0.07	P>0.05

There was no effect of d62 MT-TP on insulin concentrations (d90 female vehicle n = 6, MI-TP n = 8). Pancreatic tissue was collected from control and MI-TP-exposed female fetuses, enzymatically dispersed and cultured in vitro. Only tissue from vehicle treated pregnancies was able to mount the expected increase in insulin secretion (*P<0.05) in response to elevated (20 mM) glucose in vitro. Under euglycaemic glucose concentrations (5.5 mM), in vivo MI-TP-exposure lead to a significantly reduced SI (#P<0.05) in female fetuses, however, basal insulin secretion from MI-TP fetal pancreatic tissue was significantly elevated as compared to tissue derived from vehicle treated fetuses (P<0.05), and comparable to maximal secretion of vehicle treated tissue stimulated by 20 mM glucose.

### Maternal Androgen Treatment also Increases Fetal Estradiol Concentrations

**Figure 5:**
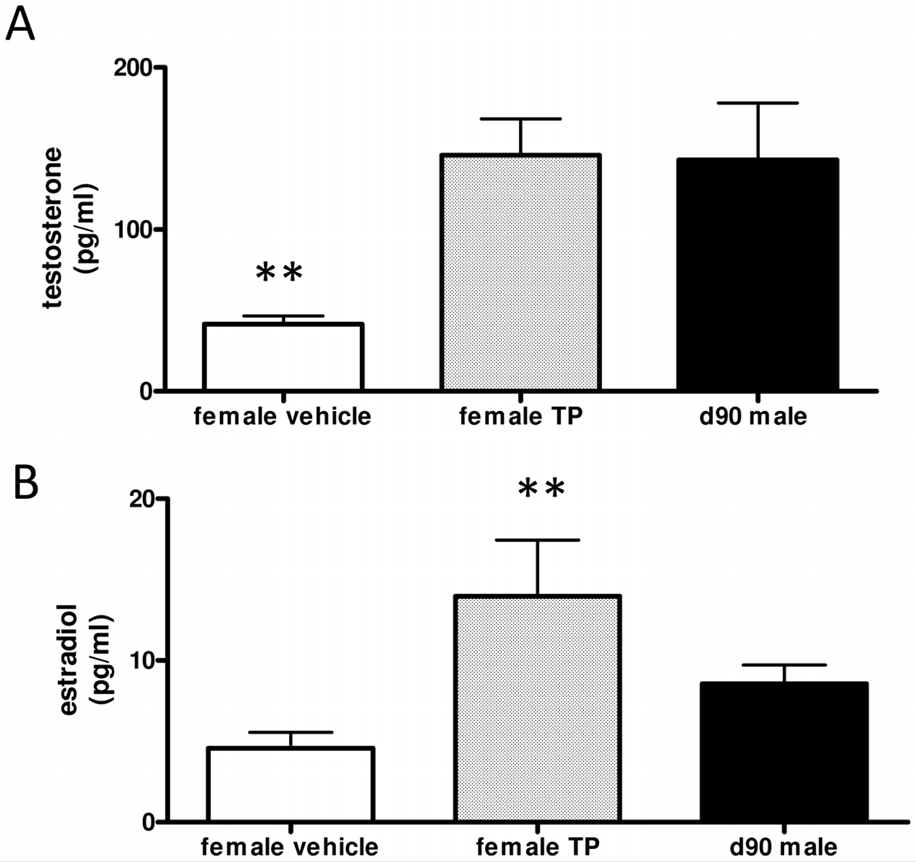
Fetal plasma sex steroid concentrations. In female fetuses, d62 MI-TP administration increased testosterone concentrations significantly, up to levels similar to that found in male fetuses (d90 vehicle vs TP, *P*<0.01). Female plasma estradiol was significantly increased by d62 MI-TP treatment at d90 gestation (*P*<0.05) beyond that of male fetal plasma (d90 female vehicle n = 6, MI-TP n = 8; male vehicle n = 10).

Maternal d62 MI-TP administration raised fetal female testosterone concentrations at d90 to that seen in male fetuses (*P*<0.05; Fig. 5A). However the placenta can aromatize androgen to estrogen and fetal estradiol concentrations were also significantly (P<0.05) greater than vehicle treated female fetuses after maternal androgenisation (Fig. 5B).

### Pancreatic Effects of Direct Injection of Androgens and Estrogens into the Fetus

**Figure 6:**
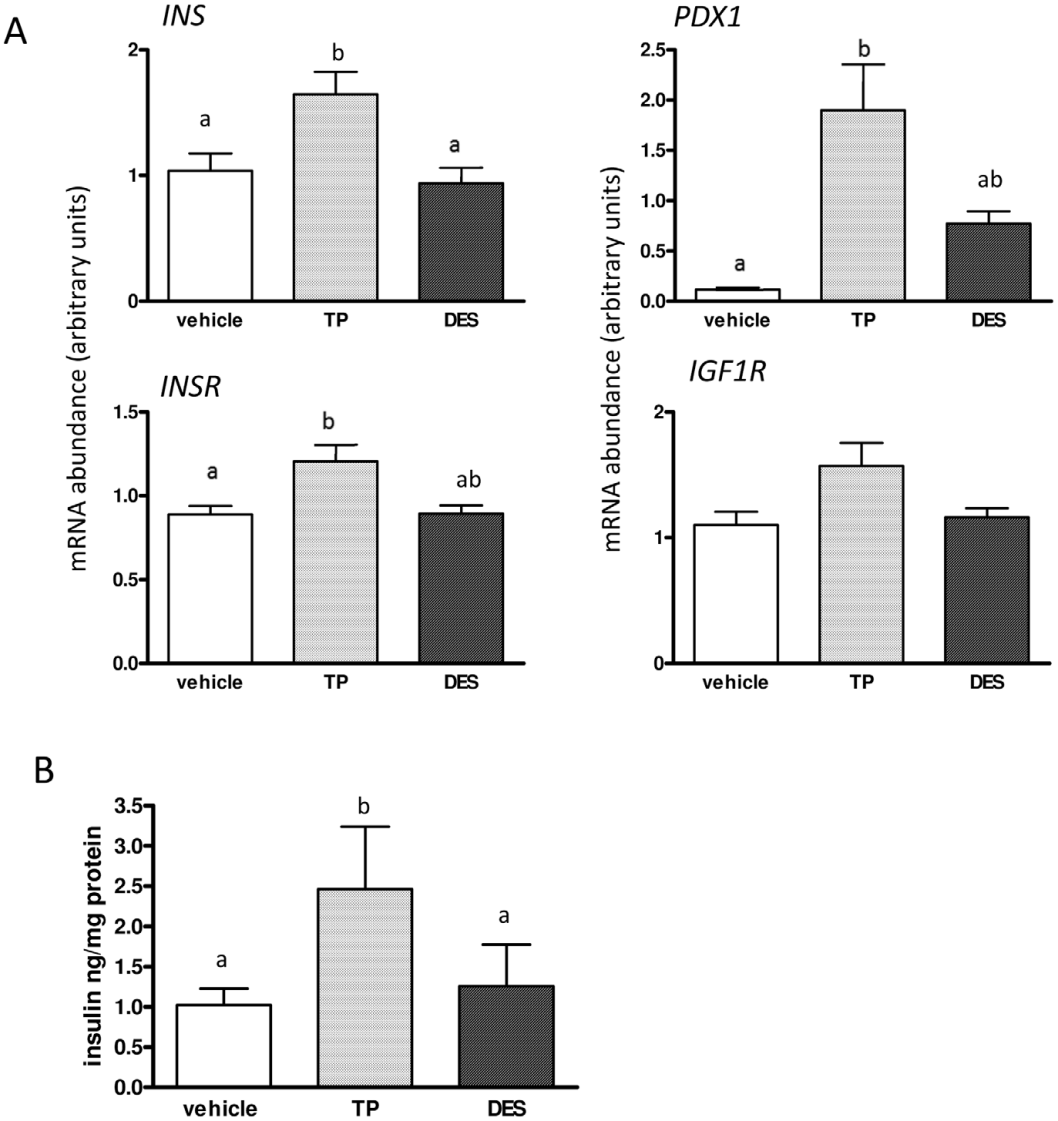
Comparison of direct, fetal administration of TP and DES: effects on pancreatic gene expression, β-cell counts and *in vitro* insulin secretion. To delineate androgenic effects of MI-TP administration from potential estrogenic effects attributable to metabolism of applied androgen to estrogen, TP or the estrogen agonist DES were directly applied to female fetuses (FI-TP/FI-DES) and tissue collected at d90 of gestation. Genes whose expression was significantly altered in response to MI-TP (Panel A) were analysed (vehicle n = 6, FI-TP n = 7, FI-DES n = 5), *in vitro* insulin secretion in response to euglycaemic conditions (5.5 mM glucose) was determined (Panel C) (vehicle n = 5, FI-TP n = 7, FI-DES n = 4). *INS*, *PDX1* and *INSR* were significantly elevated by FI-TP (*P*<0.05), but not FI-DES. As regards *IGF1R*, there was a trend towards increased expression associated with FI-TP but not FI-DES (*P* = 0.06). FI-TP, but not FI-DES was associated with elevated insulin secretion *in vitro* in response to euglycaemic culture conditions as compared to *in vivo* vehicle treated cultures (*P*<0.05).

To dissect the effects of androgens and estrogens we directly injected either TP, DES or vehicle into the fetus at d62 and d82 of gestation and analysed the effects at d90 gestation. Pancreatic genes whose expression was altered in female fetuses at d90 after d62 MI-TP treatment were examined after direct fetal treatment. We found that FI-TP, but not FI-DES administration, caused significant increases in the expression of *INS* (P<0.05), *PDX1* (*P*<0.05), *INSR* (*P*<0.01), and a strong trend in the case of *IGF1R* (*P* = 0.06) (Fig. 6A). In addition *in vitro* cultures of tissue recovered from the pancreas of fetal sheep directly injected with either TP or DES showed an increased secretion of insulin in direct response to FI-TP, but no difference as compared to control cultures in the case of DES, in response to a fixed, euglycaemic concentration (5.5 mM) of glucose (*P*<0.05) (Fig. 6B).

## Discussion

Maternal administration of exogenous androgens during pregnancy perturbs reproductive and metabolic function of offspring in various species during adult life, the clinical relevance being that this adult physiology, in both reproductive and metabolic aspects, is reminiscent of PCOS in women [4], [6], [9], [10], [19]. PCOS encompasses metabolic abnormalities such as IR and, as a downstream consequence, hyperinsulinemia in women [2], [5], which are recreated by *in utero* overexposure to androgens in animal models.

In studies of both *in utero* androgen overexposure animal models, and human PCOS, there are intriguing indications of pancreatic β-cell dysfunction [3], [20], and suggestions that the causal relationship between hyperinsulinaemia and IR, at least in lean women with PCOS, is weaker than perhaps commonly assumed [21]. In a previous study [9] we observed that prenatal androgenization via maternal administration of TP commencing at day 62 of gestation gave rise to offspring that exhibited early hyperinsulinemia, raising the question as to whether or not IR underpinned increased insulin secretion, or there was a primary pancreatic alteration.

Examination of the possibility of pancreatic changes occurring in response to an altered *in utero* steroid milieu is pertinent in light of findings that the pancreas is a sex-steroid responsive tissue [6], [22], [23], and observations of altered β-cell function in early postnatal life in a mouse model of *in utero* androgenic exposure [6]. Studies utilizing primate androgen exposure models also are suggestive of β-cell alterations, evident in early post-natal life in terms of increased insulin secretion in response to glucose challenge. Whilst the explanation for this was persistence of a fetal response to maternal hyperglycaemia [7], programming effects of androgens directly on the pancreas may also be possible.

Analysis of gene expression and protein phosphorylation relevant to insulin signalling in target tissues was undertaken to assess if we could detect molecular peripheral insulin resistance in the MI-TP offspring sheep that demonstrate an increased insulin response to glucose [9]. This is in light of reports of altered insulin signalling in PCOS, via altered Ras-ERK activity [15], and elevated Akt-mediated insulin signalling, associated with IR and hyperinsulinaemia [24]. We detected no alterations in insulin signalling at the molecular level in the target tissues we analyzed. This, with the early hyperinsulinemia [9] raised the possibility of primary pancreatic alterations as a consequence of prenatal androgen overexposure. In a similar ovine model where androgen exposure began at d30, hyperinsulinemia in offspring was observed, but could not be definitively ascribed to IR [25].

It should be noted that the majority of previous studies of the ovine adult phenotype created by prenatal androgenization commenced maternal androgen treatment at d30 gestation [25], [26], [27] and the PCOS-like changes [28], [29] that occur after starting later have been less well studied. However commencement of TP administration at d62 means that female offspring develop normal external female genitalia while displaying alterations in ovarian follicular gene expression commensurate with alterations in thecal steroidogenesis found in women with PCOS [10] and early metabolic imbalance with hyperinsulinemia [9]. Interestingly, studies in primate models showed that earlier gestation androgen excess was possibly associated with impaired β-cell function [30], indicative that timing of exposure and cross-species comparisons must be carefully considered.

Examination of pancreatic tissue from 11 month old adult offspring from our d62 MI-TP androgen overexposed pregnancies revealed increased β-cell numbers, and increased numbers of β-cells per islet, suggesting that fetal androgen overexposure may have had direct effects that persisted into post-natal life. In women with PCOS it has been suggested that β-cell dysfunction is permanent [31], and rodent models show differential responses to elevated glucose at 3–6 months postnatal age from androgen treated pregnancies [6]. We detected no alteration in the islet α-cell content of these adult pancreases, and in this regard it is pertinent to note that studies of effects of maternal nutrition on pancreatic development in sheep fetuses reveal plasticity of β-cell development, but not α-cell development [32]. We therefore focussed attention on examining fetal pancreatic β-cell responses to over-exposure to androgens.

Ovine fetal pancreatic β-cells expressed androgen receptors, suggesting androgen responsiveness. AR immunoreactivity was also noted in non-insulin positive cells in pancreatic islets, in line with previous work in the mouse pancreas where islet cells *per se* expressed AR [23], indicative that androgens may exert effects on other endocrine cells of the pancreas.

We observed no alterations in fetal plasma insulin due to treatment (or fetal sex), but this is coloured by glucose concentrations and clearance, such that it is to a large extent maternal/placental regulated, and therefore it is not straightforward to ascribe fetal plasma concentrations of insulin directly to fetal β-cell function/secretory capacity. However, when fetal pancreatic tissue was challenged *in vitro* by elevating glucose concentrations from 5.5 mM (normal pregnant ewe glucose is approximately 3–5 mM, [33]) to 20 mM, pancreatic tissue derived from androgen-exposed female fetuses did not mount an insulin response whereas tissue from vehicle-treated female fetuses demonstrated the expected rise in insulin secretion. Interestingly, *in vitro* insulin secretion was higher in response to 5.5 mM glucose in TP-exposed female tissue than in female controls, comparable to the insulin output realised by 20 mM glucose in female tissue and subsequently *in vitro* SI was reduced by TP treatment. Prenatally androgenized mouse islets show impaired glucose sensing, and secreted approximately twice the amount of insulin that control islets secreted in response to moderate, euglycaemic glucose concentrations [6], consistent with our observations in the ovine model.

The pancreas of PCOS patients with a family history of type 2 diabetes mellitus (T2DM) has a somewhat limited capacity for increased insulin secretory response to glucose [34]. This has also been observed in obese adolescents with PCOS, suggesting greater risk of T2DM development when inherent β-cell impairment, in terms of plasticity of insulin secretion, is coupled with IR [20], and more recent studies also suggest possible alterations in β-cell responsiveness to IR associated with PCOS [35].

Women with PCOS have greater insulin secretion in response to IR than women with similar IR but no PCOS [3]. Furthermore, it has also been determined that whilst weight reduction in PCOS has a favourable outcome in terms of reduction of insulin resistance, hyperinsulinemia was not reduced to the same degree [36]. Given correlations between insulin secretion and bioavailable testosterone [3], a primary pancreatic defect tipping the balance towards over-secretion of insulin may be an important factor in the development and progression of PCOS.

In order to examine potential mechanisms underpinning altered β-cell function in response to prenatal androgenization, pancreatic expression of genes with critical roles in fetal islet development and β-cell function were examined at d90 of gestation. Local expression of genes which play regulatory roles in both β-cell development and function, such as *IGF1* and *IGF2*, were unaffected by d62 MI-TP treatment in female fetuses while *IGF1R* increased. Although not β-cell specific, given the important role of IGF signalling in islet development and β-cell function [13], [37], and the similar elevated expression response to MI-TP in terms of *INSR*, it is likely that IGF and insulin signalling are potential mediators of androgen action in the pancreas. It is also noteworthy that female offspring from d62 MI-TP pregnancies had increased hepatic expression of IGF-1 [9].

*INS* expression was also elevated in response to d62 MI-TP and it was accompanied by a functional effect in the form of the aforementioned increased *in vitro* basal insulin secretion. This represents a β-cell specific effect of d62 MI-TP on the pancreas of female fetuses. Testosterone has been previously shown to increase *INS* expression [38] and be anti-apoptotic in male rat islets *in vitro* [39] although there are reports of sex-specific differences in such androgen responses [40]. A second β-cell specific gene expression alteration in response to treatment was observed in terms of *PDX1*, whose expression was significantly increased by d62 MI-TP administration.

Given the presence of AR in β-cells, and elevated testosterone concentrations in MI-TP female fetuses, these effects are likely to be due to a combination of direct androgenic stimulation of β-cells, and/or indirect effects mediated via increased growth factor receptor expression. IGF/insulin signalling can regulate β-cell mass upstream of PDX1 [41] as evidenced by reductions in IGF/insulin signalling being associated with decreased *PDX1* expression [37]. Mechanistically, islet hyperplasia and apoptotic protection has been linked to increased IGF2 signalling in rodents [17], [42]. IGF-signalling (specifically IGF2) is thought to be anti-apoptotic in β-cells (acting through IGF1R; [13]) and is suggested to prevent the post-natal wave of β-cell apoptosis in rodents [43].

In addition to its role in β-cell mass regulation [13], PDX1 also appears to play a role in glucose-driven *INS* expression [44], hence there is likely a functional relationship between increased *IGF1R, PDX1* and *INS* expression and increased *in vitro* secretion of insulin, in the d62 MI-TP female fetuses at d90. Whilst it has been suggested that glucocorticoids have a regulatory role in *PDX1* expression [45], to our knowledge this is the first time that androgenic regulation, either direct or indirect, has been observed.

Regarding the potential for long-term consequences of such gene expression alterations in fetal life, fetal growth restriction (FGR) in rodents permanently reduces *PDX1* in β-cells via altered gene methylation, culminating in adult diabetes (reviewed by [37]). In sheep, FGR is associated with altered β-cell function postnatally, and altered pancreatic expression of *INSR* and *IGF2* [13]. Collectively, these findings indicate that pancreatic expression/function of local and intrinsic regulators of β-cell replication and function can be permanently affected during *in utero* life, with consequences for adult health. Given the role of PDX1 in β-cell replication, and elevated fetal *INS* expression, these alterations in expression in response to d62 MI-TP may therefore represent fetal antecedents of the increased β-cell numbers and hyperinsulinemia observed in offspring from MI-TP manipulated pregnancies.

An overarching finding of the gene expression analysis undertaken was that MI-TP treatment, commencing at d62 of gestation, does not appear to cause development of a phenotypically ‘male’ pancreas in a female fetus, in terms of either pancreatic gene expression responses (*INS, PDX1, IGF1R* and *INSR*) to exogenous androgen exposure, or endogenous *SLC2A2* expression. In other genes examined in the d62 model there were no sex-specific differences in expression in vehicle treated samples; MI-TP effects, where evident in female fetuses, bore no apparent relationships to male expression patterns. MI-TP (d62) had no effects on male pancreatic gene expression measured at d90. Thus there remains the possibility that this lack of response is at least partially attributable to pre-exposure of the developing male pancreas to endogenous testicular androgens from commencement of testicular steroid production at d30–35 of gestation [46], [47].

It may therefore be hypothesized that this earlier exposure may drive the ‘default pancreas’ towards a ‘male’ phenotype. To address this possibility we performed an experiment where androgen exposure (MI-TP) was commenced at d30 in female fetuses to coincide with the onset of testicular steroidogenesis in males. Whilst *IGF1R* was similarly elevated in response to TP administration at either d30 or d62 of gestation in female fetuses, it was noted that β-cell associated gene expression, measured at d90, showed no response, or a muted, non-significant response, to TP administration when TP exposure began at this earlier d30 stage as compared to d62, mirroring the lack of response observed in d62 MI-TP male fetuses. This suggests that during sexual differentiation, future pancreatic sensitivity to androgenic steroids may be differentially set in males and females. Thus, females derived from pregnancies where androgen exposure commenced at d62 show the response of a female fetus to exogenous androgen exposure, whereas ovine models commencing androgen treatment at d30 may give rise to female animals which appear to be phenotypically male in terms of β-cell response to androgen exposure. This observation highlights how both early and mid-gestational TP exposure models can be utilized collectively to tease apart the effects of timing of androgen exposure during gestation, and identifies a window, in terms of downstream pancreatic response to androgen overexposure, of pancreatic sensitivity to androgens between d30 and d62 of gestation.

Female children whose mothers suffer from PCOS have elevated umbilical vein testosterone concentrations similar to concentrations observed for male babies at birth [48]. Fetal androgen concentrations in our d60 MI-TP treated female fetuses sheep were, at d90, within a range previously observed in male sheep fetuses [49] and were comparable to those observed in male fetuses in the current study. Thus the androgen-exposure regimen achieved over-exposure of female fetuses to testosterone in both a physiological, and human PCOS-relevant context.

Of additional interest was the increased concentration of estradiol observed in female fetuses at d90 in response to d62 MI-TP. Expression of estrogen receptors α and β in pancreatic β-cells has been reported and associated, via ERα [50], with increased insulin synthesis in the absence of effects on β-cell mass [51], [52]. Whilst we were unable to detect expression of ERβ by PCR, we did detect ERα expression in pancreatic tissues (data not shown). Given prior suggestions that effects of testosterone on fetal development could potentially be either androgenic in nature or via metabolism to estrogens [53], experiments to resolve androgenic from estrogenic mechanisms of action were conducted. Direct fetal steroid injection studies demonstrated that the gene expression effects observed in the MI studies of androgen action could be replicated by injection of TP into the fetus, but not by administration of DES. Similarly, in terms of *in vitro* insulin secretion, we observed elevated insulin secretion in response to euglycaemic glucose concentrations in pancreatic cultures derived from TP treated fetuses as compared to control fetuses, but no alterations in the DES treated fetal tissue. We therefore ascribe the effects seen in the MI-TP studies to androgenic stimulation as opposed to estrogenic effects.

As prenatal androgenization results in a metabolic phenotype reminiscent of PCOS [4] it is perhaps likely that it shares developmental similarities with the molecular programming of PCOS. If this ovine model, and other animal models, inform us about elements involved in development of PCOS, then it may be that insulin hypersecretion is an inherent primary feature of PCOS as previously suggested [3], [21]. We therefore propose that prenatal androgenic stimulation of pancreatic β-cell function/mass regulators may predispose to hyperinsulinemia in response to normal glucose concentrations in the absence of IR. Subsequently, compensation in response to developing IR may be altered, and that this has relevance to human PCOS. In this context it is fascinating to note suggestions of potential contributions to IR development made by increased insulin concentrations [24].

In conclusion, we have demonstrated altered development of the ovine endocrine pancreas in terms of altered expression of genes which determine β-cell function and mass, altered insulin secretory response *in vitro*, and altered β-cell numbers in early adult life, as a consequence of physiologically relevant androgen over-exposure *in utero*. We suggest that β-cell dysfunction may be distinct from altered pancreatic function in response to insulin resistance and that its origins reside in fetal life. In addition the suggestion of ‘male’ and ‘female’ tissues may be of interest in the context of stem cell biology and transplantation.
